# Dual role of microglia in health and disease: pushing the balance toward repair

**DOI:** 10.3389/fncel.2015.00051

**Published:** 2015-02-19

**Authors:** Raquel Ferreira, Liliana Bernardino

**Affiliations:** Brain Repair Group, Health Sciences Research Centre, University of Beira InteriorCovilhã, Portugal

**Keywords:** microglia, inflammation, neuronal repair, neuron-glia crosstalk, neurodegenerative diseases

Microglial cells have been traditionally regarded as the rowdy foot soldiers of the Central Nervous System (CNS), always on the edge of causing massive destruction when provoked. Nevertheless, microglia are responsible and/or strongly contribute to the maintenance of CNS homeostasis, immune surveillance and synaptic plasticity. Until now, it was believed these complex processes depended on two polarizing stimuli received by microglia: the “bad” ones leading to a classical pro-inflammatory response (M1), and the “good” ones leading to a typical anti-inflammatory profile (M2). However, under M1 and M2 polarizing conditions, microglia can *differentiate* into different population subsets and develop specific patterns of activity. Gertig and Hanisch (2014) extensively review the heterogeneity of microglia responses and discuss how it impacts cytokine production, clearance of tissue debris, antigen presentation or the ability to sense neurotransmitters. The challenge will be the development of tools that allow us to select a subpopulation of interest and modulate its response specifically to an optimal therapeutic effect. Microglia are classically addressed as the resident macrophages of the CNS. In that sense, these cells are strongly committed to removal of foreign/infectious particles and clearance of cellular components to maintain a healthy brain parenchyma. Nau et al. (2014) distinguish two pathways by which microglia increase phagocytosis and intracellular elimination of pathogens albeit with different outcomes on neuronal survival. Stimulation of one or several toll-like receptors or nucleotide-binding oligomerization domain-containing protein 2 receptors leads to the release of pro-inflammatory products causing neuronal damage. Conversely, authors report that microglia activation by palmitoylethanolamide increases phagocytosis and intracellular elimination of pathogens without the release of pro-inflammatory mediators. The discovery of molecules that can eliminate pathogens without damaging surrounding neuronal tissue will likely strengthen the ability of the brain to resist infection. On the latter phagocytic function of microglia, Gitik et al. (2014) show that paxillin and cofilin activation promotes phagocytosis of degenerated myelin in SIRP-alpha knocked-down microglia, and show a positive correlation between paxillin and cofilin activation and phagocytosis. Myelin breakdown occurs following traumatic axonal injury and neurodegenerative disorders such as multiple sclerosis. Understanding the mechanisms underlying the removal of degenerated myelin will allow us to develop the necessary strategies to promote remyelination more efficiently while preserving nearby intact tissue. This study further emphasizes the importance of studying microglia responses and the nature of their interactions with other cell types at different stages of disease progression in order to maximize overall tissue recovery and/or survival. Chronic microglia activation also occurs in amyotrophic lateral sclerosis (ALS), the most aggressive form of adult motor neuron degeneration. Brites and Vaz (2014) extensively review the intricate crosstalk between motor neurons and glial cells, including microglia, and how the latter modulate ALS onset and progression. In an early stage of the disease microglia have a beneficial effect while overly activated and pro-inflammatory microglia in the later stages of the disease become neurotoxic. The study of neuron-microglia interactions and the selection of the adequate model to study microglia contribution to this dynamics warrants attention from Neiva et al. (2014). The group discusses important biochemical and physiological differences between immortalized microglia cell lines and primary microglia and alert to possible pitfalls when using artificial culture systems. On this note, authors suggest that supplementation of microglial culture media with fractalkine induces a more *in vivo*-like typical morphology and behavior compared to most *in vitro* models. Although the proposed model requires additional characterization it is important to be aware of the strategy we choose to validate microglia therapeutic or detrimental properties. An emerging target for the development of anti-neuroinflammatory strategies is histamine, mainly known for its role as a peripheral inflammatory mediator. Rocha et al. (2014) review current literature on histamine modulation of microglia activity but also present new data on how the secretome of histamine-stimulated microglia promotes dopaminergic neuronal death. By examining the effects of antihistamines on dopaminergic cell survival, authors unveil new perspectives on therapeutic platforms for Parkinson's disease. Microglia activity changes not only in a pathological context but also in the healthy aging brain. To better understand these mechanisms, Caldeira et al. (2014) propose an *in vitro* model of reactive and aged microglia. In their study, authors report that 16 days *in vitro* microglia evidence morphological and reduced inflammatory and functional activity corresponding to irresponsive/senescent cells. An increase in life expectancy and age-related neurodegenerative diseases highlights the importance of developing tools to control and recover microglia activity in the aging brain. Both aging and neurodegenerative disorders lead to neuronal loss albeit at different extents. An approach to delay or halt neuronal death and/or to promote the replacement of dead/dying neurons is to stimulate neurogenesis. In this sense, the role of microglia on the modulation of the neurogenic niche has been increasingly addressed. Marshall et al. (2014) show how regional differences (neurogenic niches vs. cerebral cortex) can affect microglia proliferative ability in the brain. Authors conclude that microglia possess intrinsic and spatially-restricted characteristics that allow them to function as distinct populations independently of their *in vitro* environment. This distinction allows for microglia isolated from the neurogenic niches to be more efficient in promoting neurogenesis. Microglia not only modulate the neurogenic niche but interact and shape the activity of mature neurons. Cristovao et al. (2014) comment on the novel role of microglia on pre-synaptic differentiation as a means to better understand the formation of aberrant synapse formation occurring in neurodevelopment disorders. Authors show that activated microglia cause a significant increase in the axonal density of pre-synaptic marker synapsin I, which is not observed in the presence of non-primed microglia or in isolated axons. This study emphasizes the importance of dissecting microglia responses to maternal infection during pregnancy and how they may impact fetal synapse formation. In the present research topic, researchers presented their work and views on the cellular and molecular mechanisms developed by microglia in response to injury and disease, neuronal remodeling and aging. We expect the work presented herein can advance our understanding on microglia-mediated responses toward neuronal survival and regeneration and therefore promote the development of new therapeutic approaches to efficiently repair the diseased and/or injured CNS.

## Conflict of interest statement

The authors declare that the research was conducted in the absence of any commercial or financial relationships that could be construed as a potential conflict of interest.
